# Compatible and Incompatible Mycorrhizal Fungi With Seeds of *Dendrobium* Species: The Colonization Process and Effects of Coculture on Germination and Seedling Development

**DOI:** 10.3389/fpls.2022.823794

**Published:** 2022-03-10

**Authors:** Guang-Hui Ma, Xiang-Gui Chen, Marc-André Selosse, Jiang-Yun Gao

**Affiliations:** ^1^School of Ecology and Environmental Science, Yunnan University, Kunming, China; ^2^Institute of International Rivers and Eco-Security, Yunnan University, Kunming, China; ^3^Institut de Systématique, Évolution, Biodiversité, Muséum National d’Histoire Naturelle, Sorbonne Universités, Paris, France

**Keywords:** *Dendrobium*, ecological specificity, fungal co-culture and monoculture, orchid mycorrhizal fungi, plant symbiotic interactions, symbiotic seed germination

## Abstract

Orchids highly rely on mycorrhizal fungi for seed germination, and compatible fungi could effectively promote germination up to seedlings, while incompatible fungi may stimulate germination but do not support subsequent seedling development. In this study, we compared the fungal colonization process among two compatible and two incompatible fungi during seed germination of *Dendrobium officinale.* The two compatible fungi, i.e., *Tulasnella* SSCDO-5 and Sebacinales LQ, originally from different habitats, could persistently colonize seeds and form a large number of pelotons continuously in the basal cells, and both fungi promoted seed germination up to seedling with relative effectiveness. In contrast, the two incompatible fungi, i.e., *Tulasnella* FDd1 and *Tulasnella* AgP-1, could not persistently colonize seeds. No pelotons in the FDd1 treatment and only a few pelotons in the AgP-1 treatment were observed; moreover, no seedlings were developed at 120 days after incubation in either incompatible fungal treatment. The pattern of fungal hyphae colonizing seeds was well-matched with the morphological differentiation of seed germination and seedling development. In the fungal cocultural experiments, for both orchids of *D. officinale* and *Dendrobium devonianum*, cocultures had slightly negative effects on seed germination, protocorm formation, and seedling formation compared with the monocultures with compatible fungus. These results provide us with a better understanding of orchid mycorrhizal interactions; therefore, for orchid conservation based on symbiotic seed germination, it is recommended that a single, compatible, and ecological/habitat-specific fungus can be utilized for seed germination.

## Introduction

Orchids show a series of floral characteristics distinct from those of most other flowering plants. One of the characteristics is the large number of dust-like seeds, which are among the smallest seeds of all flowering plants and characterized by a small and undeveloped embryo not surrounded by an endosperm (Arditti and Ghani, 2000). Due to the lack of nutrient reserves, orchids highly rely on mycorrhizal fungi for mineral and carbon resources to germinate into seedlings under natural conditions (Smith and Read, 2008; Dearnaley et al., 2016). Orchid mycorrhizal fungi (OMFs) belong to the so-called rhizoctonias, a polyphyletic group of fungi belonging to Tulasnellaceae and Ceratobasidiaceae (in the order Cantharellales), as well as Serendipitaceae (in the order Sebacinales; Smith and Read, 2008; Dearnaley et al., 2012; Weiß et al., 2016). These taxa are not mycorrhizal in other plants, with the exception of some of their subclades (Selosse et al., 2021).

During germination, it is commonly known that orchid seeds are colonized by one or more mycorrhizal fungi to form protocorms (Leake, 1994; Smith and Read, 2008). The protocorms, known as undifferentiated seedlings or the first seedling stage of orchids, are rootless and acotyledonous conical to spherical bodies that are non-photosynthetic and heavily mycorrhized (Rasmussen and Rasmussen, 2014). New leaves and roots are formed successively from the meristematic domain at the anterior end of the protocorm (Fang et al., 2016). One of the interesting questions during seed germination is how an orchid selects its fungal partners. Orchids may associate with a wide range of mycorrhizal fungi, but their occurrence is bounded by specific habitat conditions, which are related to ecological specificity (Porras-Alfaro and Bayman, 2007; Herrera et al., 2019). Recently, increasing studies have demonstrated that in the seed germination stage, orchids require more specific mycorrhizal associations, and incompatible fungi may stimulate germination *per se* but did not support subsequent seedling development (Bidartondo and Read, 2008; Zi et al., 2014; Rasmussen et al., 2015; Rafter et al., 2016; Huang et al., 2018; Meng et al., 2019a). Symbiotic fungi undergo a taxonomic and functional bottleneck during orchid seed germination, and the interaction between partners, rather than intrinsic fungal traits, may be involved in the bottleneck of fungal symbionts during orchid seed germination (Meng et al., 2019b).

In our previous studies on *Dendrobium* species, based on fungal inoculation experiments, we found that compatible fungi were able to quickly promote seed germination up to the seedling stage, while incompatible fungi could stimulate seed germination and form protocorms, but did not support subsequent seedling development (e.g., *Dendrobium aphyllum*, Zi et al., 2014; *Dendrobium devonianum*, Huang et al., 2018; *Dendrobium moniliforme*, Meng et al., 2019b; *Dendrobium exile*, Meng et al., 2019c; *Dendrobium officinale*, Wang et al., 2021). In *D. officinale*, compatible fungi from different sources showed relative effectiveness in promoting seed germination and seedling growth (Tan et al., 2014; Zhang et al., 2020; Wang et al., 2021), and similar results were also obtained in other orchid species (Fuji et al., 2020; Kendon et al., 2020). Although there is mounting evidence that orchid seeds are often colonized by multiple fungi simultaneously, most *in vitro* germination experiments focus on mycorrhizal monocultures, and less is known about how mycorrhizal assemblages affect the seed germination and growth of seedlings (Shao et al., 2020; Wang et al., 2021). The mycorrhizal symbioses between orchids and fungi might be influenced by inherent differences among the closely related fungi, and the symbiotic mechanisms are still unclear but may involve fungal effector and plant receptor genes similar in plant-pathogen interactions (Favre-Godal et al., 2020; Fuji et al., 2020).

It is well known that fungal hyphae grow into orchid tissues through the suspensor cells of seeds during the interactions and form elaborate coiled structures known as pelotons within cortical cells (e.g., Uetake and Peterson, 1997; Peterson et al., 2004). Because fungal compatibility is relative to orchid species and a fungus is compatible with an orchid but may be incompatible with another orchid species, we are curious about what differences occur during the colonization process between compatible and incompatible fungi. In this study, we used a well-studied medicinal orchid, *D. officinale*, to compare the fungal colonization process among different compatible and incompatible fungi during seed germination. We also tested the effects of cocultures of compatible and incompatible fungi on seed germination and seedling development in *D. officinale* and *D. devonianum*. We hope these results provide a better understanding of orchid mycorrhizal interactions and aid future studies on symbiotic mechanisms.

Here, we presented our results concerning two aspects of symbiotic interaction, namely, (1) What are the differences of colonizing process to seeds between compatible and incompatible mycorrhizal fungi in *D. officinale*? and Do incompatible mycorrhizal fungi form pelotons in orchid seeds or protocorms? and (2) What are the effects of cocultures on germination and seedling development in *D. officinale* and *D. devonianum*? and Do cocultures of two compatible fungi increase protocorm formation and seedling development and do cocultures of one compatible fungus with one incompatible fungus reduce protocorm formation and seedling development?

## Materials and Methods

### Orchid Seeds and Mycorrhizal Fungi

Seeds of two *Dendrobium* species, *D. officinale* and *D. devonianum*, were used in this study. We conducted and assisted outcross-pollination trials on cultivated plants of two orchids during March and April 2017, and the close-to-dehiscent fruits of *D. officinale* and *D. devonianum* were harvested in November 2017 and April 2018, respectively. Seeds were dried and stored in the Orchid Seed Bank of Yunnan University using the same methods described by Gao et al. (2014). Prior to each use, seeds were tested using the 2,3,5-triphenyltetrazolium chloride (TTC) method to ensure high viability (>96%; Vujanovic et al., 2000).

Four OMFs, namely, *Tulasnella* SSCDO-5, Sebacinales LQ, *Tulasnella* FDd1, and *Tulasnella* AgP-1, were used in this study to compare their effects on the seed germination of *D. officinale* and *D. devonianum*. The original source, compatibilities with *D. officinale* and *D. devonianum* based on our previous studies, and the related information about the four OMFs are summarized in Table 1. The living cultures of all four fungal strains were deposited in the Lab of Ecology and Evolutionary Biology, Yunnan University, China. In addition, the strain of *Tulasnella* sp. FDd1 was also deposited in the China General Microbiological Culture Collection Center (accession number: CGMCC No. 9551), and the strain of Sebacinales LQ was deposited in the China Center for Type Culture Collection (accession number: CCTCC No. M2019744).

**TABLE 1:** The four orchid mycorrhizal fungi (OMFs) used in this study with their original source, compatibilities to *D. officinale* and *D. devonianum*, and effects on seedling formation of host orchid species.

OMFs	Compatibility		original source of fungi	Fungal effects on seedling formation of host orchid species	Genbank accession number	References
					
	*Dendrobium officinale*	*D. devonianum*				
*Tulasnella* sp. SSCDO-5	Yes	No	Protocorms of *D. officinale*	The seedling formation rate was 41.10 ± 3.9% at 90 days after incubation.	MH348614	Shao et al., 2019
Sebacinales LQ	Yes	Yes	Protocorms and roots of *D. officinale*	The seedling formation rate was 70.09 ± 3.2% at 90 days after incubation.	MN173026	Wang et al., 2021
*Tulasnella* sp. FDd1 (*Epulorhiza* sp. FDd1)	No	Yes	Protocorms of *D. devonianum*	The seedling formation rate was 72.36 ± 11.7% at 50 days after incubation.	KM226996	Huang et al., 2018
*Tulasnella* sp. AgP-1 (*Tulasnella* sp. 2)	No	No	Protocorms of *Arundina graminifolia*	The seedling formation rate was 79.75 ± 3.84% at 35 days after incubation	MK651838	Meng et al., 2019a

### Orchid Seeds Incubated With Compatible and Incompatible Orchid Mycorrhizal Fungi

Seeds of *D. officinale* were inoculated with compatible fungi (e.g., SSCDO-5 and LQ) and incompatible fungi (e.g., FDd1 and AgP-1) to compare seed germination and the morphological differences in fungal hyphae colonizing seeds during the process of symbiosis. Seeds were sterilized with 1% (w/v) sodium hypochlorite solution (NaClO) for 5 min and washed with sterile distilled water 3–5 times. Using a pipette, *ca*. 30 seeds, suspended in 1 ml of agar solution, were transferred into a Petri dish containing 20 ml of sterile oatmeal agar (OMA; 4 g/L) medium adjusted to a pH of 5.6 before autoclaving. Once the seed was transferred, each Petri dish was inoculated with 1 cm^3^ of fungal inocula placed in the center of the Petri dish for each of the SSCDO-5, LQ, FDd1, and AgP-1 treatments, and two control treatments without a fungal strain were carried out on OMA medium (nutrient-poor medium) and MS medium (nutrient-rich medium; Murashige and Skoog, 1962). Each treatment was replicated in 30 Petri dishes that were placed in illumination incubators (RXZ300B, Ningbo Southeast Instrument Co., Ltd., Ningbo, China) under the conditions of 25 ± 2.0°C and a 12-h/12-h light/dark cycle.

The stages of seed germination for each treatment were monitored and recorded regularly. At 30, 60, 90, and 120 days after incubation, seed materials from all treatments that accurately represented the most common stage in the treatment group (e.g., ungerminated seeds, germinated seeds, protocorms, or seedlings) were sampled and stained, following the methods described by Phillips and Hayman (1970). Seed samples were cleaned using 10% potassium hydroxide (KOH) solution at 90°C for 30 min, bleached with 3% hydrogen peroxide solution, washed with 1% hydrochloric acid (HCl) solution, stained in 0.05% (w/v) trypan blue in lactic acid glycerol solution at 37°C for 30 min, and then destained in acetic glycerol solution overnight. The statuses of fungal hyphae colonized with seed materials were observed under the microscope (DM2000, Leica Microsystems GmbH, Wetzlar, Germany).

### Effects of Fungal Cocultures on Seed Germination and Seedling Formation

To compare the effects of fungal cocultures on seed germination and seedling formation, seeds of *D. officinale* and *D. devonianum* were inoculated with one compatible fungus, one incompatible fungus, two compatible fungi, and one compatible fungus with one incompatible fungus. Approximately, 30 sterilized seeds were sown on OMA medium in each Petri dish, and each Petri dish was inoculated with two 1 cm^3^ pieces of fungal inocula. A total of six fungal treatments for *D. officinale* and five fungal treatments for *D. devonianum* were finally performed, and OMA and MS media without fungal inoculation were also used as nutrient-poor and nutrient-rich control treatments for two orchid species, respectively (Table 2). Each treatment was replicated with 30 Petri dishes and placed in an illumination incubator at 25 ± 2°C and a 12-h/12-h light/dark cycle.

**TABLE 2:** The fungal strains used in different fungal cocultural treatments with seeds of *D. officinale* and *D. devonianum*.

Treatments	Fungi strains co-cultured with seeds of two orchids	
	*Dendrobium officinale*	*D. devonianum*
Single compatible fungus	**LQ**	**LQ**
	**SSCDO-5**	**FDd1**
Single incompatible fungus	FDd1	SSCDO-5
One compatible and one incompatible fungi	**LQ** + FDd1	**FDd1** + SSCDO-5
	**SSCDO-5** + FDd1	\
Two compatible fungi	**LQ** + **SSCDO-5**	**FDd1** + **LQ**
Nutrient-poor control treatment	OMA medium without fungal strain	
Nutrient-rich control treatment	MS medium without fungal strain	

*The black bold of fungal codes represents the compatible fungal strain.*

According to the results of our previous studies, the LQ strain could quickly promote seed germination of *D. officinale* and seedling formation in the LQ treatment approached the peak level at 60 days after incubation (Wang et al., 2021), while for *D. devonianum*, seedling formation in the FDd1 treatment reached the maximum at 50 days after incubation (Huang et al., 2018). Therefore, in this study, the number of seeds and the stages of seed germination were assessed and recorded at 60 days after incubation for each Petri dish. We adopted the following criteria of Arditti (1967): Stage 0, non-germination; Stage 1, embryo swells and turns green, and testa is popped open (germination); Stage 2, continued embryo enlargement forms a spherule and seed coat broken (protocorm formation); Stage 3, appearance of protomeristem (protocorm differentiation); Stage 4, seedling with the emergence of the first leaf; and Stage 5, emergence of second leaf and further development.

### Data Collection and Statistical Analysis

We used Stages 0, 1, 2 + 3, and 4 + 5 to determine no germination, seed germination, protocorm formation, and seedling development, respectively. Total seeds (*t*), germinated seeds (*g*), protocorms (*p*), and seedlings (*s*) were counted at 60 days after incubation. The percentages of germinated seeds (*G*), protocorms (*P*), and seedlings (*S*) were calculated as follows: *G* = 100 × (*g* + *p* + *s*)/*t*, *P* = 100 × *p*/*t*, and *S* = 100 × *s*/*t*, respectively.

The one-sample Kolmogorov-Smirnov test, a non-parametric test, was used to test whether the data in each group obeyed a normal distribution. One-way ANOVA and least significant difference (LSD) in cases when data were subjected to a normal distribution, and generalized linear models (GLMs) when data did not meet normal distribution were used to test the effects of different fungal inoculations on seed germination, protocorm formation, and seedling development for the two orchid species, respectively. All statistical analyses were performed in SPSS (version 25.0). The results are expressed by mean ± standard error (SE), and the alpha-type I error was fixed at 5% (thus, all non-significant differences have *p* > 0.05).

## Results

### Orchid Seeds Incubated With Compatible and Incompatible Orchid Mycorrhizal Fungi

**FIGURE 1:**
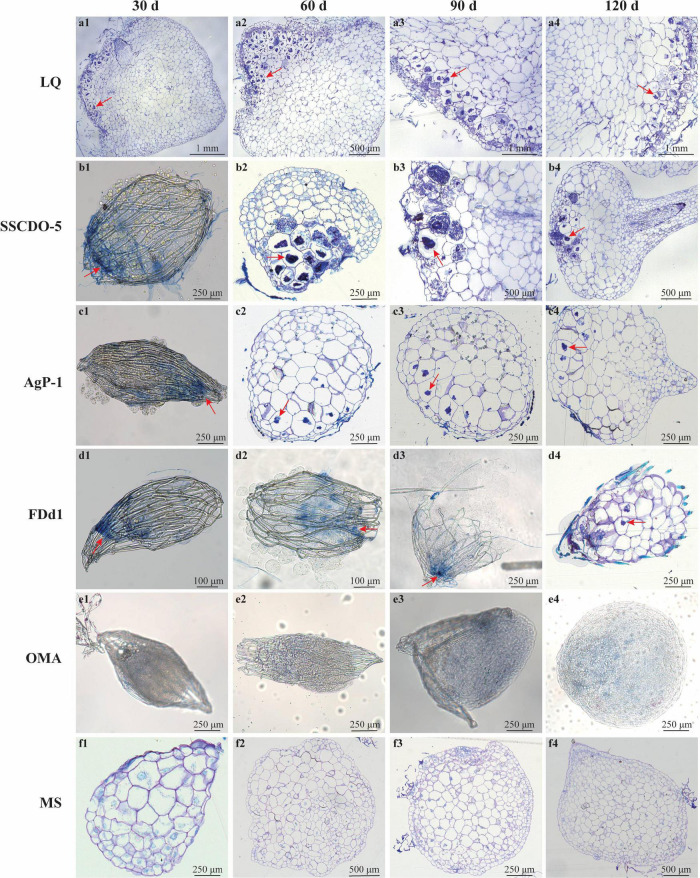
Morphological characteristics of hyphae of compatible or incompatible strains incubated with *D. officinale* seeds at different times, and cross-sections of protocorms and seedlings showing pelotons. **(a1–a4)** Seeds incubated with compatible LQ; **(b1–b4)** seeds incubated with compatible SSCDO-5; **(c1–c4)** seeds incubated with incompatible AgP-1; **(d1–d4)** seeds incubated with incompatible FDd1; **(e1–e4)** seeds on oatmeal agar medium (nutrient-poor medium); **(f1–f4)** seeds on MS medium (nutrient-rich medium). The red arrows indicate pelotons inside the cells of protocorms or seedlings, or, the positions that the fungal hyphae clustered on the seed surfaces and into the seeds.

Overall, seed germination, protocorm formation, and seedling development of *D. officinale* showed great differences between compatible fungal treatments and incompatible fungal treatments. The compatible fungi, SSCDO-5 and LQ, were capable of quickly promoting seed germination up to the seedling stage, while no seedlings were found in the incompatible fungal treatments inoculated with AgP-1 or FDd1 strains until 120 days after incubation (Figure 1).

#### Thirty Days After Incubation

In the LQ treatment, most of the seeds had already formed protocorms (Stage 2 or 3) and shed seed coats, and a large number of pelotons in the basal cells of the protocorm could be observed (Figure 1a1). In the SSCDO-5 treatment, most seeds turned green with embryos swollen (Stage 1), fungal hyphae congregated at the basal end outside of seeds, and a few pelotons in the basal cells of enlarged seeds were also observed (Figure 1b1). Meanwhile, in the AgP-1 and FDd1 treatments, all seeds remained ungerminated (Stage 0), but some hyphae congregated at the basal end outside of seeds, and scattered pelotons were occasionally found in the basal cells of seeds (Figures 1c1,d1). For the two control treatments, seeds in the OMA treatment were ungerminated (Stage 0; Figure 1e1), while seeds in the MS treatment were enlarged with embryos swollen (Stage 1; Figure 1f1), and this situation was similar to the seeds in the SSCDO-5 treatment.

#### Sixty Days After Incubation

In the LQ treatment, protocorms had differentiated into seedlings with the first leaf (Stage 4), and a large number of pelotons occurred inside the cells of the protocorm staining black-blue (Figure 1a2 and Supplementary Figure 1A2). In the SSCDO-5 treatment, seed embryos constantly expanded, the seed coat broke from the chalazal end, and most of the seeds reached the protocorm stage (Stage 2 or 3), and many pelotons had been formed in the basal cells of the protocorm (Figure 1b2). At this stage, protocorms (Stage 2) also occurred in the AgP-1 treatment, and the situation was very similar to those of the SSCDO-5 treatment (Figure 1c2). However, seeds in the FDd1 treatments were still in the ungerminated stage (Stage 0), although hyphae were observed entering inside the seeds (Figure 1d2). Meanwhile, seeds in the OMA treatment were still in Stage 0 (Figure 1e2), but most of the seeds in the MS treatment formed protocorms at Stage 2 or 3 (Figure 1f2).

#### Ninety Days After Incubation

At this stage, seedlings with two leaves (Stage 5) were the common status in the LQ treatment, and pelotons occurred inside the most cells of the protocorm (Figure 1a3 and Supplementary Figure 1A3). The situation in the SSCDO-5 treatment was very similar to the LQ treatment (30 days earlier), and seedling bore the first leaf (Stage 4) and a large number of pelotons were observed (Figure 1b3). In the AgP-1 treatment, most of the seeds had already reached the protocorm stage (Stage 3), and some pelotons had been formed in the basal cells of the protocorm (Figure 1c3 and Supplementary Figure 1C3). In the FDd1 treatment, seeds expanded slightly but were still in Stage 0, and more hyphae surrounding the seed could be observed (Figure 1d3 and Supplementary Figure 1D3). In the OMA treatment, seeds began to germinate, and the seed coats of many seeds had already broken from the chalazal end (Stage 1; Figure 1e3), while most of the seeds had already formed protocorms (Stage 2 or 3) in the MS treatment (Figure 1f3).

#### One Hundred and Twenty Days After Incubation

Seedlings continuously developed and bore 3–4 leaves (beyond Stage 5), and interestingly, the number of pelotons was observed to be obviously reduced in plant cells compared to 30 days earlier (Figure 1a4). In the SSCDO-5 treatment, most seedlings bore two leaves (Stage 5), and massive pelotons occurred inside the cells (Figure 1b4). For two incompatible fungi, protocorms remained unchanged (Stage 3) and no seedlings were found in the AgP-1 treatment (Figure 1c4), while in the FDd1 treatment, a few seeds started to germinate with seed coat broken (Stage 1), and some fungal hyphae could be seen at the basal end outside of seeds, but no pelotons were found inside the seeds (Figure 1d4 and Supplementary Figure 1D4). In the OMA treatment, the status of seeds remained unchanged at Stage 1 (Figure 1e4), while some protocorms started to differentiate the first leaf (Stage 3 or 4) in the MS treatment (Figure 1f4).

### Effects of Fungal Cocultures on Seed Germination and Seedling Formation

**FIGURE 2:**
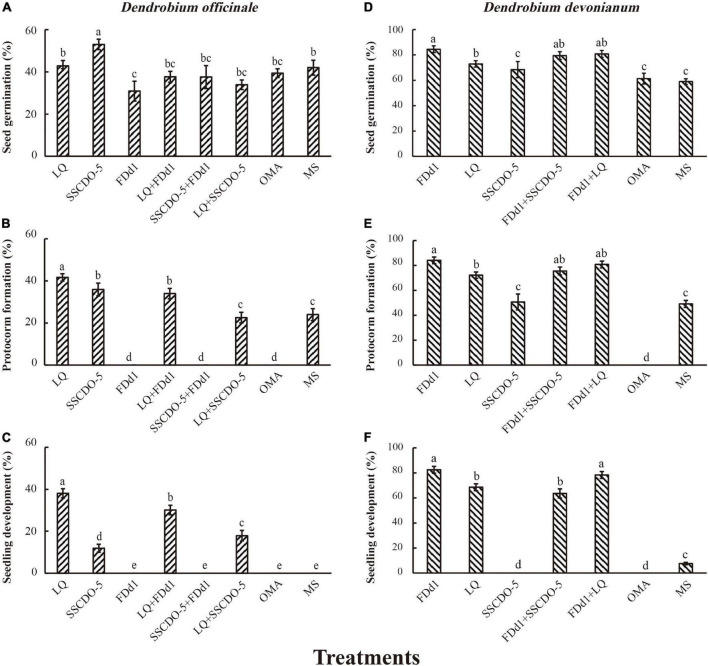
Statistical analysis results of different mixed-fungal treatments at three stages of seed development. The different strains incubated with *D. officinale* seeds **(A–C)**; the different strains incubated with *D. devonianum* seeds **(D–F)**; the significance among fungal treatments is shown by different lowercase letters at *p* < 0.05.

At 60 days after incubation, seed germination, protocorm formation, and seedling development showed similar patterns for both orchid species. No seedlings were formed in incompatible fungal treatments or OMA treatments, and the cocultures of seeds with two compatible fungi and with one compatible fungus and one incompatible fungus had negative impacts on seedling development (Figure 2).

#### For *Dendrobium officinale*

The percentage of seed germination was significantly higher in the SSCDO-5 treatment (53.00 ± 2.46%) than in the other treatments (all *p-*values < 0.05), and the lowest seed germination (30.89 ± 4.73%) occurred in the FDd1 treatment (Figure 2A). However, protocorm formation varied greatly among different treatments. No protocorms were found in the FDd1, SSCDO-5 + FDd1, and OMA treatments, and the percentage of protocorm formation in the LQ treatment (41.67 ± 1.74%) was significantly higher than that in any other treatment (Figure 2B). At this stage, no seedlings were found in the FDd1, SSCDO-5 + FDd1, OMA, and MS treatments. For the two compatible fungal treatments, the percentage of seedlings in the LQ treatment (38.11 ± 2.16%) was significantly higher than that in the SSCDO-5 treatment (11.89 ± 1.93%; *p* < 0.01), while the percentages of seedlings in the two cocultural treatments were all significantly lower than those in the LQ treatment but significantly higher than those in the SSCDO-5 treatment. Interestingly, the percentage of seedlings in the cocultural LQ + FDd1 treatment with one compatible fungus and one incompatible fungus (30.11 ± 2.27%) was significantly higher than those in the two compatible fungi of LQ + SSCDO-5 treatment (17.89 ± 2.49%; *p* < 0.01), while no seedlings were formed in the cocultural SSCDO-5 + FDd1 treatment (Figure 2C).

#### For *Dendrobium devonianum*

The percentage of seed germination was significantly higher in the FDd1 treatment (84.44 ± 2.70%) than in the LQ, SSCDO-5, and two control treatments (all *p-*values < 0.05), but had no significant differences with the two cocultural treatments of FDd1 + SSCDO-5 (*p* = 0.33) and FDd1 + LQ (*p* = 0.48), respectively (Figure 2D). Protocorms were formed in all treatments except for the OMA treatment, and the percentage of protocorm formation showed similar patterns to seed germination (Figure 2E). However, no seedlings were found in the SSCDO-5 and OMA treatments. For the two compatible fungal treatments, the percentage of seedlings in the FDd1 treatment (82.44 ± 2.78%) was significantly higher than that in the LQ treatment (68.56 ± 2.75%; *p* < 0.01). For the two cocultural treatments, the percentage of seedlings in the FDd1 + SSCDO-5 treatment with one compatible fungus and one incompatible fungus (63.56 ± 3.64%) was significantly lower than that in the two compatible fungi of the FDd1 + LQ treatment (78.33 ± 2.72%; *p* < 0.01) and FDd1 treatment, respectively. Although the percentage of seedlings in the FDd1 + LQ treatment was lower than that in the FDd1 treatment, there was no significant difference between the two treatments (*p* = 0.21). The percentage of seedlings in the MS treatment was 7.56 ± 0.93%, which was significantly lower than that in the other four treatments (Figure 2F).

## Discussion

Orchids have complex symbioses with fungi throughout their lifespan, and OMFs may have important impacts on the distribution, abundance, and population dynamics of orchid populations, and are particularly important for the key processes, such as seed germination and seedling establishment (Jacquemyn et al., 2012; McCormick et al., 2018; Shefferson et al., 2020). Orchids recruit OMFs from rhizoctonias that are often considered to live as saprobes in the soil around the roots (Smith and Read, 2008) or on tree bark around epiphytic orchids (Petrolli et al., 2021). The Waiting Room Hypothesis proposed that OMFs were recruited from ancestors that colonized roots as endophytes, and root endophytism in the orchid family was a predisposition for mycorrhizal evolution (Selosse et al., 2009, 2021). Therefore, host-fungal compatibility may be influenced largely by environmental factors (Smith and Read, 2008).

The genus *Dendrobium* Swartz is one of the largest genera in Orchidaceae, and many species in the genus have been used as traditional Chinese medicine (TCM) with a very long history in China (Bao et al., 2001). Of those, *D. officinale* is undoubtedly the most popular medicinal species with the largest scale of massive commercial cultivation in China (Cheng et al., 2019); therefore, it has received much research attention regarding its mycorrhizal symbioses including isolation and identification of effective symbiotic fungi for seed germination and plant growth (see review by Teixeira da Silva et al., 2015). *D. officinale* is a lithophytic or epiphytic orchid widely distributed in subtropical areas, including southwestern Anhui, western Fujian, northwestern Guangxi, Sichuan, Taiwan, southeastern Yunnan, and eastern Zhejiang in China, and grows in different habitats, e.g., growing on trees in forests, on rocks in karst landforms, and on sandy conglomerates in Danxia landforms under different vegetation types (Wang et al., 2021). Different studies have reported fungi from different resources with a significant favorable ability to promote seed germination and seedling development in *D. officinale* (e.g., Wang et al., 2011, 2021; Chen et al., 2012; Tan et al., 2014; Shao et al., 2019; Zhang et al., 2020), making it an ideal species for the studies of mycorrhizal symbioses between orchid and OMFs.

In this study, the process of fungal colonizing seeds during germination in *D. officinale* was observed and compared among two compatible and two incompatible fungi. In the early stage of symbiosis, fungal hyphae gathered at the suspensor of the seeds and colonized the plant cells from the suspensor. Rasmussen (1995) speculated that if original colonization occurs through the suspensor cells before seed germination, then the mycorrhizal fungi could promote seed germination. Meng et al. (2019a) also found that two fungi of *Tulasnella* penetrated the seeds of *Arundina graminifolia* from suspensor cells, and promoted seed germination. Although seeds of *D. officinale* could germinate in all four fungal treatments (at Stage 1), they showed great differences in subsequent development among treatments. In the LQ treatment, seeds could quickly germinate, form protocorms, and develop into seedlings accompanied by a large number of pelotons formed inside the cells of protocorms, while in the SSCDO-5 treatment, seed germination, protocorm formation, and seedling development were much slower than in the LQ treatment, and also fewer pelotons were obviously observed in different stages compared with the LQ treatment. In two incompatible fungal treatments, although most seeds reached the protocorm stage (Stage 3) and a few pelotons were also observed at 90 days after incubation, no seedlings were found even at 120 days after incubation in the AgP-1 treatment, while in the FDd1 treatment, only a few seeds started to germinate with seed coat broken (Stage 1), and no pelotons were observed at all 120 days after incubation.

The results showed that the pattern of fungal hyphae colonizing seeds was well-matched with the morphological differentiation of seed germination and seedling development, and compatible fungi could persistently colonize seeds and quickly promote the conversion of germinated seeds into seedlings. The number of pelotons in protocorms has been used as a proxy for successful symbiosis, and it can be positively correlated with protocorm growth in mycorrhizal symbioses in many orchids (e.g., Hadley and Williamson, 1971; Masuhara and Katsuya, 1989; Fuji et al., 2020). It was suggested that the symbiotic compatibility is regulated separately between seed development and subsequent protocorm growth and implies a critical role for the latter in overall orchid fitness (Fuji et al., 2020).

In this study, three phylogenetically related *Tulasnella* strains differed greatly in colonization modes. *Tulasnella* SSCDO-5 is symbiotically compatible, while *Tulasnella* AgP-1 and FDd1 are symbiotically incompatible with *D. officinale.* The two compatible fungi, *Tulasnella* SSCDO-5 and Sebacinales LQ, were isolated from protocorms of *D. officinale* originally from different habitats, and both could promote seed germination up to seedling with relative effectiveness. Because OMFs have been recruited among endophytic fungi that colonize orchid ancestors (Selosse et al., 2021), the host-fungal compatibility may be influenced by environmental factors, which was referred to as ecological specificity and considered to be critical in evaluating the ecological consequences of compatibility in mycorrhizal associations (Smith and Read, 2008; Põlme et al., 2018; Kendon et al., 2020).

In the fungal cocultural experiments, for both orchids of *D. officinale* and *D. devonianum*, cocultures had slightly negative effects on seed germination, protocorm formation, and seedling formation. The results are consistent with those of other studies (Sharma et al., 2003) and have also been observed in other *Dendrobium* species, e.g., *Dendrobium nobile*, for which it was also found that cocultures did not result in significantly higher seed germination percentages and seedling development than monocultures (Shao et al., 2020). In arbuscular mycorrhizal fungi, when two species of the same genus (*Acaulospora*) were cocultured with the host plant *Metrosideros laurifolia*, the biomass value was reduced when compared with the same inoculation treatment with only one species of *Acaulospora* (Crossay et al., 2019). Such reduced efficiency as a result of functional redundancy between close species was explained as induced competition (e.g., Thonar et al., 2014; Gosling et al., 2016; Yang et al., 2016).

These results suggest that there is antagonism or competition between mycorrhizal fungi for the studied species, which may be caused by the environmental conditions of the habitat. An unexpected observation was that some rhizoctonia-associated epiphytic orchids displayed ectomycorrhizal taxa, and the surprising occurrence of ectomycorrhizal fungi on tree bark suggests that their ecological niche still hides some unknown aspects (Selosse et al., 2018). OMFs have a broad geographical distribution, and similarly, orchids may associate with a wide range of mycorrhizal fungi, but their occurrence is bounded by specific habitat conditions, which is related to ecological specificity (Porras-Alfaro and Bayman, 2007; Herrera et al., 2019). Ecologically, fungi can limit the size and distribution of orchid populations because the patchy distribution of mycorrhizal fungi can affect key processes, such as seed germination, plant growth, and survival (Jacquemyn et al., 2012). However, it is unclear whether the mycorrhizal fungi are different between distribution areas, and the variation in mycorrhizal associations in orchid species is to some extent affected by specific environmental conditions. Therefore, obtaining efficient ecological/habitat-specific fungi for seed germination is of importance in the conservation practices for orchids (Reiter et al., 2018; Wang et al., 2021).

## Conclusion

During seed symbiotic germination of *D. officinale*, compatible fungi could persistently colonize seeds and quickly promote the conversion of germinated seeds into seedlings, and the pattern of fungal hyphae colonizing seeds was well-matched with the morphological differentiation of seed germination and seedling development. Two phylogenetically distant fungi, *Tulasnella* SSCDO-5 and Sebacinales LQ, originally from different habitats, were compatible with *D. officinale*, and both could promote seed germination up to seedling with relative effectiveness, indicating that the host-fungal compatibility could be influenced by environmental factors. Cocultures had slightly negative effects on seed germination, protocorm formation, and seedling development for *D. officinale* and *D. devonianum*. For orchid conservation based on symbiotic seed germination, it is recommended that a single, compatible, and ecological/habitat-specific fungus can be utilized for seed germination.

## Data Availability Statement

The original contributions presented in the study are included in the article/Supplementary Material, further inquiries can be directed to the corresponding author/s.

## Author Contributions

J-YG and G-HM designed the experiment and wrote the manuscript. G-HM and X-GC conducted the experiments. G-HM performed the analysis. J-YG and M-AS reviewed the manuscript. All authors contributed to the article and approved the submitted version.

## Conflict of Interest

The authors declare that the research was conducted in the absence of any commercial or financial relationships that could be construed as a potential conflict of interest.

## Publisher’s Note

All claims expressed in this article are solely those of the authors and do not necessarily represent those of their affiliated organizations, or those of the publisher, the editors and the reviewers. Any product that may be evaluated in this article, or claim that may be made by its manufacturer, is not guaranteed or endorsed by the publisher.

## Supplementary Material

Supplementary Figure 1 Morphological characteristics of hyphae of compatible or incompatible strains incubated with *D. officinale* seeds at different times. **(A1–A4)** Seeds incubated with compatible LQ; **(B1–B4)** seeds incubated with compatible SSCDO-5; **(C1–C4)** seeds incubated with incompatible AgP-1; **(D1–D4)** seeds incubated with incompatible FDd1; **(E1–E4)** seeds on OMA medium (nutrient-poor medium); **(F1–F4)** seeds on MS medium (nutrient-rich medium).
