# Supplementary figures and images for: Compatible and Incompatible Mycorrhizal Fungi With Seeds of Dendrobium Species: The Colonization Process and Effects of Coculture on Germination and Seedling Development
Source: Front Plant Sci. 2022 Mar 10;13:823794. doi: 10.3389/fpls.2022.823794 (PMC8961024; doi:10.3389/fpls.2022.823794)

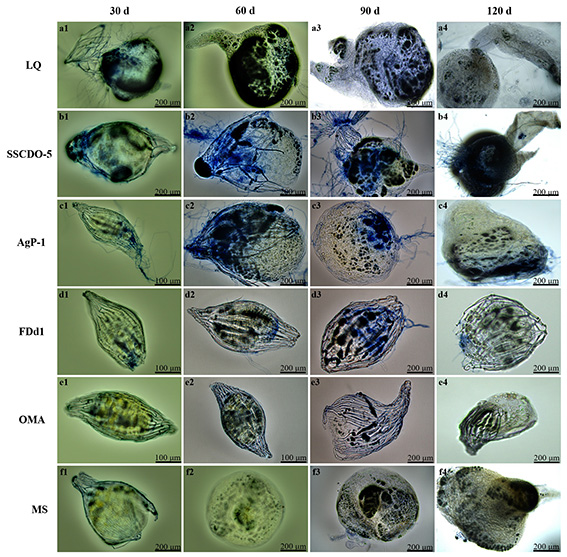

Supplement: Supplementary Figure 1 — Morphological characteristics of hyphae of compatible or incompatible strains incubated with D. officinale seeds at different times. (A1–A4) Seeds incubated with compatible LQ; (B1–B4) seeds incubated with compatible SSCDO-5; (C1–C4) seeds incubated with incompatible AgP-1; (D1–D4) seeds incubated with incompatible FDd1; (E1–E4) seeds on OMA medium (nutrient-poor medium); (F1–F4) seeds on MS medium (nutrient-rich medium). [file Image_1.JPEG]
